# Bimetallic Nanoparticles for Antimicrobial Applications

**DOI:** 10.3389/fchem.2020.00412

**Published:** 2020-05-28

**Authors:** Naman Arora, Kavitha Thangavelu, Georgios N. Karanikolos

**Affiliations:** ^1^Department of Chemical Engineering, Khalifa University, Abu Dhabi, United Arab Emirates; ^2^Center for Membranes and Advanced Water Technology (CMAT), Khalifa University, Abu Dhabi, United Arab Emirates

**Keywords:** bimetallic, antibacterial, nanoparticles, nanostructures, antimicrobial, bacteria, support, metals

## Abstract

Highly effective antimicrobial agents are needed to control the emergence of new bacterial strains, their increased proliferation capability, and antibacterial resistance that severely impact public health, and several industries including water, food, textiles, and oil and gas. Recently, bimetallic nanoparticles, formed via integration of two different metals, have appeared particularly promising with antibacterial efficiencies surpassing that of monometallic counterparts due to synergistic effects, broad range of physiochemical properties, and diverse mechanisms of action. This work aims to provide a review on developed bimetallic and supported bimetallic systems emphasizing in particular on the relation between synthesis routes, properties, and resulting efficiency. Bimetallic nanostructures on graphene, zeolites, clays, fibers, polymers, as well as non-supported bimetallic nanoparticles are reviewed, their synthesis methods and resulting properties are illustrated, along with their antimicrobial activity and potential against different strains of microbes.

## Introduction

Microbial contagious epidemics are becoming among the major causes of morbidity and mortality, with the fast spread of drug-resistant infectious diseases constituting a global threat to human health. The development of resistance to antibiotics by microbes occurs primarily through enzyme production that can alter, passivate, or deteriorate the antibiotic action, while drug resistance can also evolve through bacterial changes and adjustments of efflux pathways restricting medication passage (Davies and Davies, 2010; Magiorakos et al., 2012). Notably, it has been predicted that if the trend continues at the current speed, by 2050 more people will die because of drug-resistant infections than cancer, accounting for number of deaths in the order of 10 million (Shallcross et al., 2015; Sugden et al., 2016). In the food industry, pathogens and biofilms can proliferate on the surface of foods or packaging, compromising the quality of food products, while microbiological contamination results in high costs to food industry in terms of bacteria-impacted products that need to be withdrawn. From a technological point of view, uncontrollable bacteria proliferation/biofouling on surfaces of industrial interest, including desalination, oil and gas processing, water treatment, and food processing, cause a serious loss of productivity, compromised quality, large amounts of energy spent, and high costs.

To this extent, design of novel and highly efficient antibacterial agents is needed. Moving along this direction, there is an urgent need for fields that did not communicate with each other before, to come together in a synergistic way combining knowledge from their respective discipline toward integrated solutions that move across multidisciplinary interfaces. Nanotechnology, defined as the three-dimensional control of molecular structure to create materials and devices to molecular precision, represents such a field that can cooperate with biological and medical sciences promisingly enough to provide revolutionary nanobiology and nanomedical solutions toward fundamental understanding of existing and new diseases, diagnostics, and treatment. Metal nanoparticles (NPs) have great potential to be utilized as antimicrobial agents, such as in medical devices, wastewater treatment, food packaging, synthetic textiles, and dentistry (Zhao et al., 2011; Cheng et al., 2012; Montazer et al., 2012; Prombutara et al., 2012; Zhang et al., 2012). In general, the antimicrobial activity of metal NPs has been evaluated against various types of pathogenic bacteria, indicatively being tested against *Staphylococcus aureus, Escherichia coli, Streptococcus mutans*, and *Pseudomonas aeruginosa*, which are mainly accountable for many human epidemics (Moritz and Geszke-Moritz, 2013). These NPs typically exhibit superior performance compared to conventional antibiotics and antimicrobial treatments owing to the fact that microorganisms cannot generate resistance to them since the NPs suppress the formation of biofilm and activation of other associated processes, thus outwitting the mechanism of drug resistance. Notably, unlike conventional antibacterial agents, these nanomaterials can also be employed to carry and deliver additional antibacterial drugs, i.e., operate as drug delivery scaffolds, while they can also exhibit antimicrobial activity by themselves. Interestingly, the side toxicity of various metal-based NPs has been observed to be relatively neutral toward the environment and humans when the NP dosage is provided at an optimal level sufficient to only suppress the microbial growth (Hoseinzadeh et al., 2017).

Bulk metals or metal precursors can be converted into metal NPs, with typical sizes from 1 to 100 nm, either by top–down or bottom–up approaches. In top–down methods, bulk materials are sized down to NP size ranges through approaches, such as ball milling or attrition, often resulting in wide size distribution, non-uniform shapes, and with increased levels of contamination. Bottom–up approaches on the other side, involving procedures, such as colloidal synthesis, sol–gel processing, chemical reduction and precipitation, and atomic layer deposition, result in smaller sizes, more uniform size and shape distributions, and better control over defects and surface properties (Gates et al., 2005; Sau and Rogach, 2010; Biswas et al., 2012). NPs having smaller sizes than bacteria cells and exhibiting large surface area/volume ratios can undertake strong interplay with bacteria and exert substantial, targeted, and prolonged antimicrobial activity even at smaller dosages (Martinez-Gutierrez et al., 2010).

Metallic nanomaterials are usually found in monometallic or bimetallic formulations. Monometallic NPs are composed of only one type of metal with distinct physical and chemical properties. They can be synthesized by various approaches, mostly by chemical methods, and their surfaces can be altered by different functional moieties. In general, they are used for various applications, including in catalysis, and in optical and electronics fields. Metal NPs, and in particular silver, gold, and gallium, have exhibited unique antibacterial effects, which have been thoroughly studied against a number of microorganisms including Gram-positive and Gram-negative bacteria and viruses (Gold et al., 2018). Furthermore, noble metal NPs, such as silver and gold, in various forms have been applied in applications, such as textiles, sprays, food preservatives, dental resin composites, cosmetics, medical device coatings, implants, and medical instruments due to their strong and sustainable antibacterial action (Kassaee et al., 2008; Eby et al., 2009; Larese et al., 2009; Mohammed Fayaz et al., 2009; Pollini et al., 2009; Vasilev et al., 2009).

**Figure 1:**
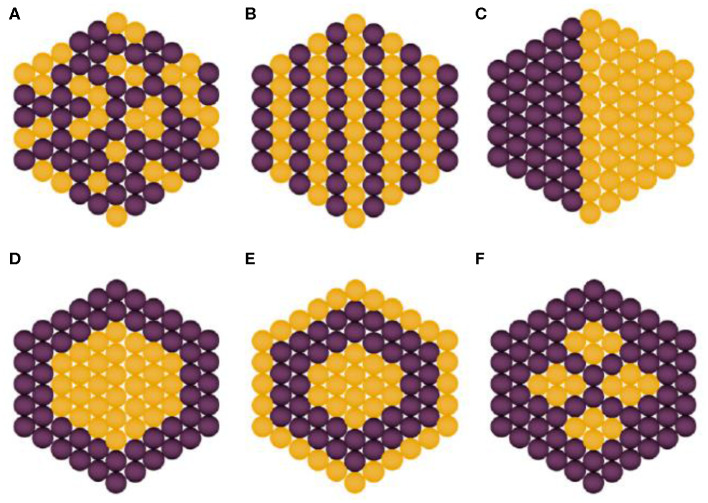
Types of bimetallic nanoparticles: **(A)** alloyed, **(B)** intermetallic, **(C)** subclusters, **(D)** core–shell, **(E)** multishell core–shell, and **(F)** multiple core materials coated by a single shell. Yellow and purple spheres represent two different kinds of metal atoms. Adapted from Srinoi et al. (2018) under the Creative Commons CC BY license.

Bimetallic NPs have acquired particular attention in the past decade in research and technological domains because of their unique optical, electronic, magnetic, and catalytic properties, which, in most of the cases, are significantly distinguishable from their monometallic counterparts. Bimetallic NPs are formed by combining two different types of metal NPs and can have a variety of morphologies and structures (Belenov et al., 2017). They typically exhibit more intriguing properties as compared to the corresponding monometallic NPs, a fact that is attributed to the synergistic properties between the two different metal parts. Tuning of properties and performance can be achieved by selecting the proper metal combination and support as well as optimizing the composition of each metal type. Bimetallic nanostructures can be categorized into two classes, namely, mixed and segregated ones, which can be further classified according to their configuration of atoms, i.e., alloy, intermetallic, subclusters, and core–shell types. In the case of mixed structures, alloyed and intermetallic particles are formed by two different metals possessing random and ordered arrangement of atoms, respectively. Unlike mixed structures, the segregated structures are typically obtained via multistep reactions, wherein the formation of an initial structure of one metal type takes place first followed by the addition of the second metal. Notably, these structures can be further classified into subcluster structures (separate distribution of two metals with a shared interface), core–shell structures (metal core is surrounded by shell of second metal), multishell core–shell structures (shells possess alternative arrangement forming a shape like onion rings), and multiple core materials coated by a single shell, as shown in Figure 1 (Ferrando et al., 2008; Srinoi et al., 2018). They are often synthesized by concurrent reduction of two metal ions with appropriate stabilization strategies, such as enabling steric hindrance and electrostatic repulsive forces. Upon synthesis, reduction rates of the two metal precursor ingredients can be controlled so as to result in desired size, shape, structure, morphology, and metal distribution of the resulting bimetallic NPs (Sharma et al., 2019). A synergistic antibacterial effect is targeted by such bimetallic NPs, and systems whose antibacterial efficiency has been recently studied include Ag–Au, Ag–Cu, Fe–Ag, and Cu–Ni (Markova et al., 2013; Argueta-Figueroa et al., 2014; Gulam Mohammed et al., 2014; Hashim, 2016; Perdikaki et al., 2016, 2018).

## Antimicrobial Action Through Different Mechanisms

**Figure 2:**
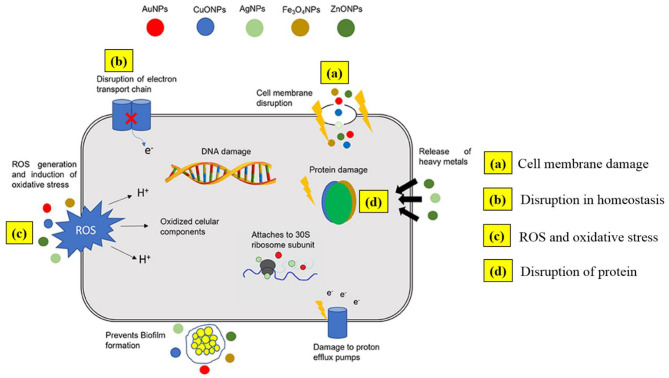
Outline of possible antimicrobial mechanisms by metal nanoparticles. Adapted from Baptista et al. (2018) under the Creative Commons CC BY license.

Metal NPs, just like antibiotics, have the ability to discern bacterial from mammalian cells because of the cells' capacity to diverge metal transport systems and metalloproteins. Metal-based NPs can personalize bacterial efficiency via various mechanisms of action (Figure 2), mainly via cell membrane degradation through electrostatic interactions, disturbance in homeostasis through protein binding, reactive oxygen species (ROS) generation and oxidative stress, disruption of proteins and enzymes, genotoxicity, and signal transduction inhibition. Retardation of bacterial growth is accomplished either by one or combination of the above mechanisms depending on the nature and chemistry of the metal NPs involved (Baptista et al., 2018). Consequently, multiaction effects are possible by combining more than one metal types in the same nanostructured system.

### Cell Membrane Degradation Through Electrostatic Interactions

The surface and spores of bacteria typically carry a negative charge at physiological pH due to the acid functional groups in proteins. Gram-negative bacteria possess higher negative charge inherently and thus manifest stronger electrostatic interaction with metal NPs than Gram-positive bacterial strains. The superior negative charge in Gram-negative bacteria is attributed to more charge per unit surface in the lipopolysaccharide lipid bilayer compared to other phospholipids (Beveridge, 1999). The electrostatic communication is thus initiated at the NP surface through interaction of positively charged NPs and negatively charged bacterial membranes. The electrostatic force of attraction is enhanced by increasing surface area, an effect that is drastically more pronounced in NPs compared to the bulk counterparts due to the increase in the ratio of external surface area per unit of mass of material upon particle size reduction. These interactions alter the structure and permeability of the cell membranes, induce film disruption, cause extended oxidative stresses, and damage bacterial proteins. Because of the breaking of cell wall, water from the cytosol is discharged, and cells attempt to compensate this via bacteria's proton efflux pumps and electron transport, a fact that promotes damage to the bacterial membrane (Nathan and Cunningham-Bussel, 2013). The resulting shortcoming of ions and suppression of membrane stability due to cell wall breaking results in decreased breath ability (suffocation), interruption of vitality transduction, and cell death (Pelgrift and Friedman, 2013). Such actions have been particularly demonstrated through involvement of metal-based NPs, such as silver, gold, zinc oxide, magnesium oxide, and titanium oxide. Silver, for instance, explicitly associates with sulfur-containing constituents of the cell membrane, and the ions produced obstruct the cell wall assembly (Kim et al., 2007). Metal NPs can also attach to mesosomes and disturb cellular respiration, affect cell division, and prevent DNA replication (Raghunath and Perumal, 2017).

### Disturbance in Homeostasis Through Protein Binding

The normal metabolic functions of bacteria are disturbed when they are exposed to excess of metal or metal ions. A main mechanism by which metal NPs exhibit antibacterial activity is via binding to cytosolic proteins, like enzymes and DNA. The positively charged metal ions enable electrostatic interactions neutralizing the negative charge on lipopolysaccharide and increasing the permeability of the outer membrane, thus contributing to the accumulation of metal ions inside the cells. The bacterial cell membrane becomes disorganized, and therefore, bacterial development is suppressed. The interaction of metal NPs with cytosolic proteins causes decreased cell function inhibiting respiratory and metabolic pathways as well as ATP production. Metal ions interact with thiol groups of the peptidoglycan layer, leading to cell wall disruption (Kim et al., 2006). Ag in particular impedes replication and division by adhering to enzyme within the respiratory chain and DNA, whereas Au binds with DNA by upregulating genes within the cell (Sondi and Salopek-Sondi, 2004; Morones et al., 2005; Zheng et al., 2017).

### ROS Generation and Oxidative Stress

Metal NPs accumulated on the bacterial cells and, with the aid of increased permeability of the outer cell membrane, promote the production of ROS, such as superoxide anions (${\text{O}}_{2}^{\cdot -}$), hydroxyl radicals (OH^·^), hydrogen peroxide (H_2_O_2_), and organic hydroperoxides. These species can be lethal to microbes, as they disintegrate bioorganic molecules, such as amino acids, carbohydrates, lipids, nucleic acids, and proteins. Important prerequisites for ROS generation are an operating redox cycle, functional moieties with oxygen groups on the nanoparticle surface, and cell–particle interplay. Electronic properties modification and decrease in molecular size generates ROS on the nanoparticle surface. Electron donor/acceptor interactions and communication with molecular oxygen enable the development of superoxide anion (${\text{O}}_{2}^{\cdot -}$), which cascades generation of more ROS via Fenton-type reactions (Nel et al., 2006). The physiochemical properties of the metal NPs, such as surface area, diffusivity, surface properties, and electrophilic nature affect the quantity of ROS formed in the bacteria cells. The generation of ROS typically inhibits respiratory enzymes (Hajipour et al., 2012; Thekkae Padil and Cerník, 2013). The free radicals peroxidize the unsaturated phospholipids in the cell membranes and produce more peroxyl radicals, promoting further membrane damage. The membrane integrity deteriorates because of oxidation of lipids leading to conformational changes in proteins of the membrane and modifying layer configuration, uprightness, and horizontal association, thus compromising membrane function. Notably, malondialdehyde, an indicator of cell membrane injury, increases due to lipid peroxidation by ROS, and ion imbalance occurs as ions flow out due to membrane malfunction (Saito et al., 1992; Ayala et al., 2014). Therefore, ROS generation causes oxidation of lipids, modification of proteins, formation of openings or pits inside the bacterial layer, hindrance of enzymes, and RNA/DNA damage, eventually leading to cell death (Sondi and Salopek-Sondi, 2004; Wang et al., 2011; Nathan and Cunningham-Bussel, 2013). Indicatively, hydroxyl radicals are mainly formed from silver, whereas gold enables ROS generation via enhanced catalytic activity producing H_2_O_2_ from glucose oxidase. Gallium has also been shown to produce ROS (Richter et al., 2017). Furthermore, glutathione, a non-enzymatic antioxidant, prevents bacteria from oxidative stress. Metal ions oxidize cellular glutathione producing excessive ROS. The oxidized glutathione also increases lipid peroxidation in the bacterial membrane, damaging the microbes via oxidative stress. To this end, combination of more than one type of metal into properly configured bimetallic NP formulations can achieve multifunctionality and enhance action via more than one mechanism, thus providing higher antibacterial efficacy.

### Disruption of Proteins and Enzymes

Another mechanism of antibacterial activity exerted by metal NPs is through protein dysfunction, according to which metal ions form protein-bound carbonyls through oxidation of the amino acid side chain (Lynch and Dawson, 2008; Aggarwal et al., 2009). The degree of carbonylation directs the degree of oxidative protein damage, and this leads to catalytic retardation in enzymes resulting into protein disintegration. Adherence to non-catalytic sites and hindrance of enzyme activity is also possible. Moreover, ions combine with the thiol groups of proteins and enzymes, making the latter inert. Basically, metal ions behave as weak acids that can react with soft bases like sulfur and phosphorous present in protein and DNA, eventually leading to cell death (Hatchett and White, 1996).

### Genotoxicity and Signal Transduction Inhibition

Metal ions also combine with nucleic acids like genomic and plasmid DNA due to their electrical properties and restrain the cell division of microbes by disturbing the reproduction of chromosomal and plasmid DNA (Giannousi et al., 2014). They also combine with 30S ribosomal subunit and hamper production of proteins from messenger RNA (mRNA). Furthermore, metal ions dephosphorylate the phosphotyrosine, an essential component in signal transduction, and this inhibition ultimately suppresses bacterial growth (Kirstein and Turgay, 2005; Shrivastava et al., 2007).

### Photocatalytic Degradation Mechanism

Photocatalysis is well-known for decomposition of chemical contaminants upon wastewater treatment as well as for its potency to suppress microbial growth. Indeed, the evolution of the reactive oxygen radicals during photocatalysis can inactivate bacteria by coenzyme oxidation, nucleic acid attack, lipids peroxidation, and degradation of cell membranes. Indicatively, TiO_2_ NP-coated metal plates were reported to inhibit the microbial growth under 50 min of UV light irradiation (Tsuang et al., 2008). The light through redox reactions, generation of free radicals, and thus ROS leads to photochemical alteration of the cell membrane, Ca^2+^ permeability, diminution in superoxide dismutase activity, damage to proteins/DNA, and abnormal cell division.

## Synthesis Routes

### Chemical Reduction

This method is the most preferred chemical approach, and it yields zero-valent metal NPs. It typically involves slow heating of the reaction ingredients, typically consisting of high-boiling solvents, precursors, and surfactants, to a sufficiently high temperature where monomer formation, accumulation, nucleation, and growth occur (Kwon et al., 2007). The reducing agents mainly used in this method are sodium borohyride, elemental hydrogen, Tollen's reagent, ascorbate, and more. Ag NPs are commonly synthesized by this method. Successive reduction is a promising technique to also synthesize core–shell bimetallic NPs. It includes the impeachment of a metal on the synthesized monometallic NPs of counter metal. The pre-synthesized monometallic NPs are ought to be chemically surrounded by the impregnated metal atoms.

### Thermal Decomposition

This approach typically comprises pyrolysis of metal precursors at high temperatures. High amount of energy is required for the bond breaking; thus, the process is endothermic in nature. The primary limitation of this method is the segregation of nanocrystal phase from the reactive phase at high temperatures.

### Electrochemical Reduction

In electrochemical processing, the propulsive force is electricity and involves electrolyte solutions with a two-electrode setup, where bulk metal is kept at anode and converted into metal clusters (Reetz and Helbig, 1994; Katwal et al., 2015). The metallic particles formed are stabilized by agents, such as tetraalkylammonium salts applied as supporting electrolyte. The electrolysis process is carried out under nitrogen atmosphere to discard dissolved oxygen. The metal cations move toward the cathode and bulk metal gets oxidized at the anode. The advantages of this method include low cost, high purity of particles, good control of particle size by optimizing current density, simplicity, and mainly potential for industrial integration. Metal-based materials, such as CuO and Cr_2_O_3_, synthesized by this method have been evaluated for their antibacterial activity.

### Sol–Gel Processing

Sol–gel processing is a wet route that primarily involves two main reaction paths, namely, condensation and hydrolysis of the precursors moieties. The characteristics of the obtained crystalline nanostructures are directed by the properties of the gel, agitation, and concentration of precursors, pH, and temperature (Ennas et al., 1998). Various bimetallic NPs, such as Au–Ag, Au–Pd, and Au–Pt are prepared by sol–gel processing. This method is advantageous, as it is simple, low cost, and effective in preparing high-quality NPs with high degree of homogeneity (Sharma et al., 2015).

### Chemical Precipitation

Chemical precipitation involves solidification from a solution by enabling formation of insoluble species or by forming supersaturated conditions. It includes conjoining of chemical reagents followed by formation of the precipitates (Sharma et al., 2016). It is a scalable technique with low levels of contamination, as it is typically carried out in single step. NPs, such as metal oxides and sulfides, e.g., ZnO and ZnS, are easily prepared by this method.

### Hydrothermal/Solvothermal Processing

The technique involves the use of increased temperature and autogenous pressure for the production of NPs. The method enables dilution and recrystallization in aqueous or other solvents/mineralizers of species that are normally non-soluble. The properties of the NPs rely upon pH, temperature, and pressure of the system, as well as the type of solvent, which is water in most of the cases, and nutrient concentrations used. The technique typically results in NPs of high crystallinity and purity compared to other wet-based methods, while it allows good control of the physical and chemical properties of the prepared NPs by properly tuning reaction conditions, such as temperature and precursor solution concentration. Drawbacks include high cost of equipment, as pressure-resistant autoclaves are typically needed, as well as safety given the autogenous pressure buildup in the reactors upon increase in the temperature. Zeolite and metal-based NPs are often synthesized by this method.

## Bimetallic Nanoparticle Systems and Their Antimicrobial Properties

### Zeolite-Supported Bimetallic Nanomaterials

Over the past few years, zeolites have gained attention as inorganic materials with potential for antimicrobial applications, since these acidic solid inorganic structures can act as hosts that carry metal ions and NPs over their surface, functioning as promising supports to exhibit antimicrobial properties. These inorganic materials typically contain Si, Al, and O atoms arranged in a three-dimensional crystalline structure forming cages and channels on a nanometer scale of well-oriented dimensions and shaping microporous frameworks (Weitkamp, 2000; Labropoulos et al., 2009; Stoeger et al., 2012a). The capability to synthesize zeolites in powder crystals (Basina et al., 2018) or in well-controlled films/membranes (Stoeger et al., 2012b) can enhance their versatility to be used as metal NP supports for various modes of antibacterial treatment operation. Ferreira et al. (2016) studied the antimicrobial properties of sodium Y-type zeolite (NaY)-supported bimetallic Zn–Ag materials against *E. coli* and *Bacillus subtilis* (bacteria species), as well as *Saccharomyces cerevisiae* and *Candida albicans* (yeast species). These materials were synthesized by employing an ion exchange method on commercial zeolite having faujasite structure with different concentrations of AgNO_3_ solution while maintaining the same concentration of Zn(NO_3_)_2_·4H_2_O, followed by calcination at 500°C for 8 h. X-ray photoelectron spectroscopy (XPS) on these materials indicated an uneven distribution of Zn^2+^ and Ag^+^ throughout the zeolite structure. Among the synthesized structures (NaY, Ag_0.010_-Y, Ag_0.025_-Y, Ag_0.050_-Y, Zn_0.05_Ag_0.01_-Y, Zn_0.05_Ag_0.025_-Y, and Zn_0.05_Ag_0.05_-Y), the highest microbial inhibition against bacteria and yeast species was achieved by Zn_0.05_Ag_0.025_-Y, with a characteristic minimum inhibitory concentration (MIC) value of 0.10 mg/ml that could be attributed to the synergistic effect between Zn^2+^ and Ag^+^ species due to their valence state and site onto the structure of zeolite.

Pereyra et al. (2014) reported a sodium A-type zeolite (NaA)-assisted Ag^+^/Zn^2+^ bimetallic material exhibiting antimicrobial action against *Aspergillus niger*. In that work, hydrothermal synthesis followed by cation exchange was employed to synthesize NaA containing different concentrations of Ag^+^/Zn^2+^, which were incorporated into waterborne coatings to investigate antifungal activity. It was observed that the zeolite sample having [Ag^+^] = 100 mg L^−1^, [Zn^2+^] = 90 mg L^−1^ achieved a growth inhibition comparable to that achieved with 230 mg L^−1^ of Ag^+1^, indicating better performance of the bimetallic over the monometallic material. Behin et al. (2016) reported Na–P zeolite loaded with Cu/Ni NPs (NaP–Cu/Ni) by employing the sonochemical method in ethylene glycol medium. The resulting material was investigated for microbial growth inhibition against *E. coli* and *B. subtilis*. In that study, NaP zeolite was obtained from natural clinoptilolite hydrothermally, followed by ultrasound-assisted modified chemical reduction to obtain the NaP–Cu/Ni material. The effect of NP loading, quantity of metal salts solution, and Cu content of NPs on the antimicrobial performance of the as-prepared material was analyzed using a Box–Behnken system. It was noticed that an approximately one-third higher antimicrobial activity was achieved by the prepared material against *B. subtilis* than against *E. coli*. Nosrati et al. (2015) synthesized a modified self-cleaning coating consisted of polyacrylic latex incorporated with Ag-exchanged zeolite A containing TiO_2_ NPs (TiO_2_-Ag-zeolite-A). This modified coating was prepared by applying solvent evaporation, and successful synthesis was confirmed by scanning electron microscopy (SEM), revealing the presence of uniformly dispersed TiO_2_ NPs of average diameter of around 30–40 nm onto the Ag-exchanged zeolite A structure. Moreover, Fourier-transform infrared spectroscopy (FTIR) confirmed the successful interaction between polyacrylic latex and TiO_2_-Ag-zeolite-A and displayed the characteristic peak at 465 cm^−1^ associated with the Ti–O bond stretching. Improved antibacterial activity against *E. coli, Listeria monocytogenes, Salmonella typhimurium, Bacillus anthracis*, and *S. aureus* was observed by the modified coating as compared to conventional grade polyacrylic latex.

Ferreira et al. (2015) investigated the antimicrobial activities of different combinations of bimetallic materials with varying concentrations of Cu, Zn, and Ag immobilized on NaY zeolite. Different combinations of monometallic and bimetallic materials were developed using an ion exchange method. Successful synthesis of one of the combinations (NaY, Ag–Y, and AgZn_0.05_-Y) was displayed by X-ray diffraction *(*XRD*)*, and subsequently, the obtained materials were evaluated for their antimicrobial properties against *E. coli* and *S. cerevisiae*. The AgCu–Y, AgZn_0.05_-Y, CuAg–Y, CuZn_0.05_-Y, Zn_0.05_ Ag–Y, and Zn_0.05_Cu–Y based structures were found to exhibit microbial growth inhibition against *E. coli* with corresponding MIC values of 1, 1, 1, >2, 0.5, and >2 mg/ml, respectively. The Zn_0.05_ Ag–Y bimetallic material attained potential antimicrobial activity with lower MIC value, attributed to a synergetic effect between Zn and Ag and their optimized concentrations. Alswat et al. employed a facile chemical method to synthesize ZnO–CuO NPs incorporated onto zeolite A, and the resulting materials were characterized by various techniques, such as SEM, XRD, FTIR, etc. (Alswat et al., 2017). SEM revealed a cubical morphology of zeolite and the presence of uniformly distributed ZnO NPs with average diameter of ~24 nm over the external surface of the zeolite, whereas CuO NPs having different shapes with diameter of ~54 nm were observed to be linked to the ZnO NPs. In addition, the diffraction peaks displayed by XRD also confirmed the cubic structure of zeolite A along with the presence of diffraction peaks associated with ZnO and CuO NPs, which were observed to be overlapped with some diffraction peaks of zeolite A. The prepared material was investigated for its antibacterial performance against microbes and was found to exhibit significant antibacterial efficacy against *B. subtilis* B29 and Gram-negative bacteria *E. coli* E266 with corresponding inhibition zones of 18.9 and 23.8 mm, respectively.

Casemiro et al. investigated the antimicrobial properties of zeolite-supported Ag–Zn nanostructures incorporated into acrylic resins (Casemiro et al., 2008). In this study, three different acrylic resins were utilized. One of them was obtained by microwave polymerization, termed as “Onda-Cryl,” while the other two were obtained by heat polymerization, termed as QC20 and Lucitone 550, respectively. The prepared resins were further modified with the introduction of different concentrations of Ag–Zn–zeolite into their matrixes and exhibited significant antimicrobial activity against *C. albicans* and *S. mutans*. The highest inhibition zone (16.78 ± 0.2) was recorded by QC20 containing 10% of Ag-Zn-zeolite against *C. albicans*. In another report, Padervand et al. employed the sol–gel process to synthesize zeolite-based Ag–AgBr, AgBr–TiO_2_, and Ag–AgBr–TiO_2_ photocatalysts that had the potential to inactivate *E. coli* (Padervand et al., 2012). It was observed that the Ag–AgBr–TiO_2_ photocatalyst exhibited better antibacterial efficacy under dark and visible light conditions. The antimicrobial activity was attributed to the generated radicals that degraded the cell wall and also to the prevention of colony formation occurred due to the interaction between DNA of the microorganisms and the released Ag^+^ ions under illumination. All reported zeolite-supported bimetallic NPs with their corresponding preparation method, inhibition zone, MIC values, and targeted microbes are listed in Table 1.

**Table 1:** Types and antimicrobial properties of zeolite-supported bimetallic nanoparticles.

**SR No**.	**Material**	**Method**	**Microbial strain**	**Inhibition zone (mm)**	**MIC (mg/ml)**	**Other performance indicators**	**References**
1	Bimetallic Zn–Ag–NaY zeolite	Ion exchange	*E. coli*/*B. subtilis*	–	0.10	–	Ferreira et al., 2016
			*S. cerevisiae*/*C. albicans*		0.30		
2	A-type Zeolite–Ag^+^ Zn^2+^	Hydrothermal	*Aspergillus niger*	–	[Ag^+^] = 100, [Zn^2+^] = 90	–	Pereyra et al., 2014
3	NaP–Cu/Ni	Sonochemical	*E. coli*/*B. subtilis*	–	–	–	Behin et al., 2016
4	TiO_2_-Ag–Zeolite-A based coating	Solvent evaporation	*E. coli*/*L. monocytogenes, S. thyphimurium*/*B. anthracis*/*S. aureus*	–	–	Suppression of CFU ml^−1^	Nosrati et al., 2015
5	AgCu–Y	Ion exchange	*E. coli*/*S. cerevisiae*	–	1/>2	–	Ferreira et al., 2015
	AgZn_0.05_-Y				1/2		
	CuAg–Y				1/>2		
	CuZn_0.05_-Y				>2/>2		
	Zn_0.05_ Ag–Y				0.5/2		
	Zn_0.05_Cu–Y based NaY zeolites				>2/>2		
6	Zeolite/ZnO–CuO	Facile chemical method	*E. coli*/*B. subtilis*	18.9/23.8	–	–	Alswat et al., 2017
7	Ag–Zn Zeolite incorporated acrylic resins	Microwave/heat-assisted polymerization	*C. albicans*/*S. mutans*	9.12–16.78	–	–	Casemiro et al., 2008
8	Ag/AgBr/TiO_2_	Sol–gel	*E. coli*	–	–	Photocatalytic degradation	Padervand et al., 2012

### Clay-Supported Bimetallic Nanomaterials

**Figure 3:**
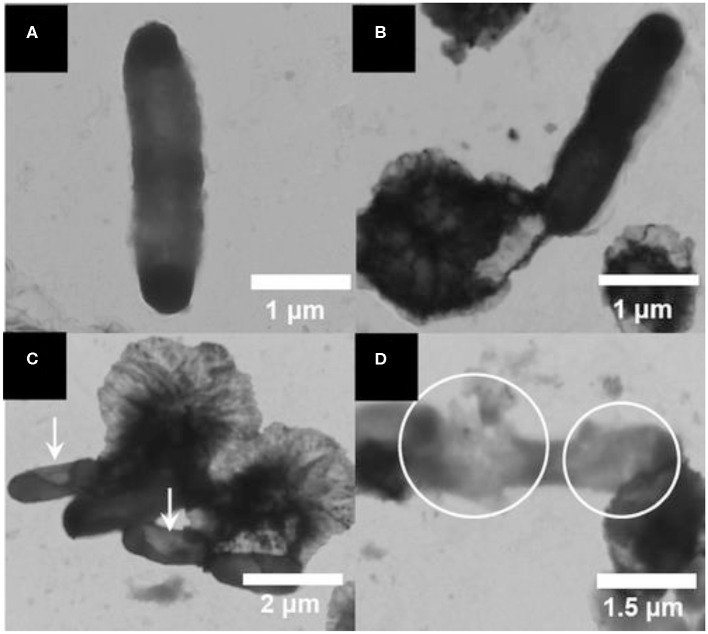
Transmission electron microscopy (TEM) images of *E. coli* photodegraded by a Ag/TiO_2_/montmorillonite (MT) composite: **(A)** *E. coli*, and *E. coli* illuminated for **(B)** 0, **(C)** 2, and **(D)** 3 h under visible light. Reproduced from Wu et al. (2010) with permission from the American Chemical Society.

A self-cleaning antibacterial coating consisting of polyacrylic latex loaded with TiO_2_/Ag^+^-exchanged montmorillonite (MT) nanocomposites (TiO_2_/Ag^+^-MT) was introduced by Olad et al. (2016). The nanocomposite fillers were prepared by dispersing TiO_2_ NPs and MT with different *w*/*w* ratios into 0.01 M of aqueous silver nitrate solution followed by agitation of this mixture for 30 h under dark conditions. The as-prepared material was recovered through filtering and washing cycles. Subsequently, the TiO_2_/Ag^+^-MT nanocomposites were introduced into a polyvinylpyrrolidone (PVP) solution, and the resulting solution was mixed with polyacrylic solution. The final self-cleaning coating was synthesized by casting 0.1 ml of obtained emulsion onto a glass substrate via solvent evaporation. Field emission SEM (FESEM), XRD, FTIR, and UV–visible characterization techniques confirmed the successful formation of fillers and the coatings. These coatings showed promising antimicrobial efficacy against both Gram-positive and Gram-negative microbial species. In another work, Wu et al. also reported montmorillonite-assisted Ag/TiO_2_ structures (Ag/TiO_2_-MT), which were prepared by applying a one-step, low-temperature solvothermal method (Wu et al., 2010). Notably, TiO_2_ NPs surrounding Ag particles were observed to be uniformly distributed over the surface of the MT clay, as revealed by TEM. The Ag/TiO_2_-MT material exhibited bactericidal activity against *E. coli* under visible light irradiation. The photodegradation of cell membrane of *E. coli* was induced by Ag/TiO_2_-MT after getting attached to it, thereby decomposing the cell membrane that leads to leakage of internal components and eventually deactivation of the bacteria. Apparent changes were noticed in the morphology of *E. coli* after illumination under visible light, which was ultimately associated to cell death, as shown in Figure 3.

A study based on Ag/TiO_2_-bentonite nanocomposite material was reported by Krishnan et al., who investigated the antimicrobial activity of this material against *S. aureus* and *E. coli* bacterial strains. In that study, a facile thermal decomposition method was applied to develop the bentonite-assisted Ag/TiO_2_ NP material that was characterized by SEM, TEM, XRD, XPS, and FTIR, revealing the successful formation of Ag/TiO_2_ NPs with an average size of 5–50 nm that were present in the interlayer sites of the bentonite clay. Importantly, the preparation of this material was carried out in the absence of reducing and precipitation agents, making the formed Ag/TiO_2_ NP clusters a product of green synthesis. Furthermore, the well-diffusion method revealed that the Ag/TiO_2_-loaded bentonite structures exhibited promising antimicrobial activity against *S. aureus* and *E. coli* with corresponding inhibition zones of 10 and 15 mm, respectively (Krishnan and Mahalingam, 2017). Shu et al. (2017) reported the synthesis of Ag and ZnO NP-decorated halloysite nanotubes (Ag–ZnO/HNTs) that exhibited synergistically enhanced antimicrobial efficacy against *E. coli*. In that work, stability and uniform dispersion of the NPs were provided by the HNTs, which also facilitated the physical contact with the cell wall of *E. coli*, enabling NPs to generate ROS in higher concentrations as well as metal ions to infiltrate the cytoplasm, causing sudden death of the bacteria. Motshekga et al. (2013) employed bentonite clay as a support toward the formation of a Ag/ZnO–bentonite composite. This material was synthesized by applying microwave-assisted synthesis that facilitated uniform dispersion of the Ag and ZnO NPs over the surface of bentonite. The resulting materials were investigated for potential antimicrobial activity against *E. coli* and *Enterococcus faecalis*. In that study, XRD revealed the presence of characteristic peaks of bentonite, observed at 2θ = 6, 20, and 35°, while the additional peaks observed at 2θ = 38° and 2θ = 32, 34, and 36° were attributed to the characteristic peaks of Ag and ZnO NPs, respectively, confirming the introduction of Ag and ZnO NPs into the bentonite clay. TEM also ensured the successful preparation of the Ag/ZnO–bentonite composite material by revealing the presence of Ag NPs (9–30 nm) and ZnO NPs (15–70 nm) onto the layers of the clay. Various compositions in the material were formulated in order to evaluate the antimicrobial efficacy. The Ag/0.4 g ZnO-clay and Ag/0.5 g ZnO-clay materials exerted the highest antibacterial performance by achieving respective corresponding inhibition zones of (7, 5, 11 mm) and (6, 7, 10 mm) against *E. coli* ATCC 11775, *E. coli* ATCC 25922, and *E. faecalis* ATCC 14506, respectively.

Motshekga et al. (2015) carried out an analogous work using bentonite as a support to avoid the aggregation of Ag and ZnO NPs, and subsequently, the resulting Ag/ZnO–bentonite composite was dispersed well in a chitosan matrix for developing Ag/ZnO–bentonite–chitosan (Cts) nanocomposites. The antimicrobial properties of the bentonite Cts nanocomposites were examined against *E. coli* and *E. faecalis*, revealing this composite as potential antibacterial agent, which exhibited a 78% bacterial removal efficiency. Ma et al. (2010) synthesized a Cu^2+^-ZnO/cetylpyridinium–montmorillonite (CZCM) material that was evaluated for its antimicrobial activity against pathogenic *E. coli* and *S. typhimurium*. The MIC values of CZCM for *E. coli* and *S. typhimurium* were recorded to be 80 and 40 μg ml^−1^, respectively, which could be attributed to the deterioration of the membrane wall of bacteria by CZCM that eventually disturbed the cellular morphology and induced the efflux of the intracellular components. In 2017, Jiao et al. (2017) synthesized a series of montmorillonite (Mt)-based composites using an ion exchange process and further loaded them with different ratios of Cu and Zn, marked as Na–Mt, Cu–Mt, Zn–Mt, Cu/Zn–Mt-1, Cu/Zn–Mt-2, and Cu/Zn–Mt-3. It was observed that Cu and Zn ions were exchanged in the interlayer space of montmorillonite, and non-uniform shapes of the particles were obtained. Furthermore, the results revealed that the antimicrobial efficiency of the Cu/Zn–Mt bimetallic material surpassed that of the monometallic (Zn–Mt and Cu–Mt) based structures, a fact that could be attributed to the synergistic effects between Cu and Zn. Among these bimetallic-based montmorillonite materials with varying Cu/Zn ratios, Cu/Zn–Mt with Cu/Zn ratios of 0.98 and 0.51 exerted higher antimicrobial efficacy against *E. coli, S. aureus*, and *C. albicans*. All reported clay-supported bimetallic NPs with their corresponding preparation method, inhibition zone, MIC values, and targeted microbes are listed in Table 2.

**Table 2:** Types and antimicrobial properties of clay-supported bimetallic nanoparticles.

**SR No**.	**Material**	**Method**	**Microbial strain**	**Inhibition zone (mm) or rate (%)**	**MIC (mg/ml)**	**Other performance indicators**	**References**
1	TiO_2_/Ag^+^-MT-based polyacrylic coating	Solvent evaporation	*L. monocytogenes*/*B. anthracis*/*S. aureus*/*E. coli/S. thyphimurium*	–	–	–	Olad et al., 2016
2	Ag/TiO_2_-MT	Solvothermal	*E. coli*	–	–	Photocatalytic degradation	Wu et al., 2010
3	Ag/TiO_2_/bentonite nanocomposites	Thermal decomposition	*S. aureus*/*E. coli*	10/15	–	–	Krishnan and Mahalingam, 2017
4	Ag–ZnO/HNTs	Precipitation	*E. coli*	–	–	Suppression of CFU ml^−1^	Shu et al., 2017
5	Ag/0.4 g ZnO-clay	Microwave assisted	*E. coli* ATCC 11775 *E. coli* ATCC 25922 *E. faecalis* ATCC 14506	7 5 11	–	–	Motshekga et al., 2013
	Ag/0.5 g ZnO-clay		*E. coli* ATCC 11775 *E. coli* ATCC 25922 *E. faecalis* ATCC 14506	6 7 10			
6	Ag/ZnO–bentonite–chitosan (Cts)	Solvent casting	*E. coli*/*E. faecalis*	–	–	Suppression of CFU ml^−1^	Motshekga et al., 2015
7	Cu^2+^-ZnO/cetylpyridinium–montmorillonite	–	*E. coli*	–	80 μg ml^−1^	–	Ma et al., 2010
			*S. typhimurium*		40 μg ml^−1^		
8	Cu/Zn–Mt-1	Ion exchange	*E. coli*/*S. aureus*/*C. albicans*	–	328.89/411.63/823.26	–	Jiao et al., 2017
	Cu/Zn–Mt-2		*E. coli*/*S. aureus*/*C. albicans*		328.89/411.63/823.26		
	Cu/Zn–Mt-3		*E. coli*/*S. aureus*/*C. albicans*		411.63/657.78/1,315.56		

### Fiber-Supported Bimetallic Nanomaterials

**Figure 4:**
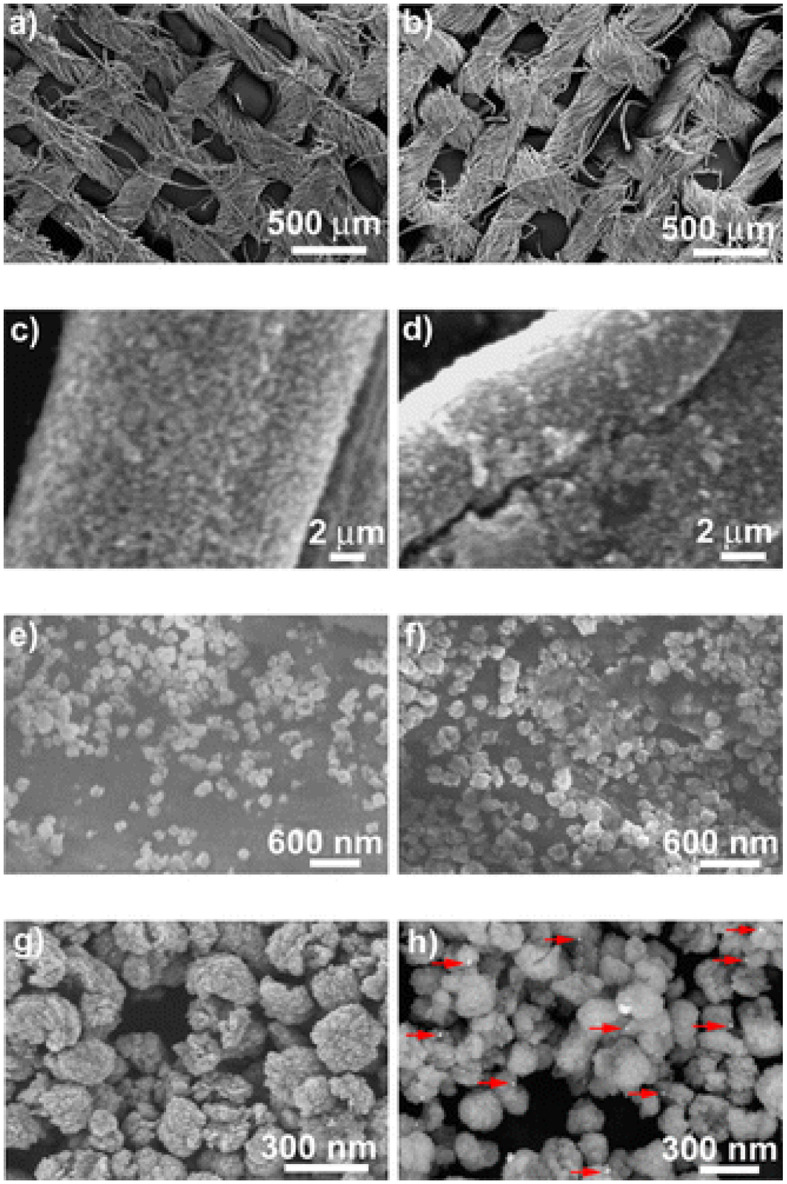
Scanning electron microscopy (SEM) images taken at different magnifications for the cotton fabrics **(a,c,e)** ZnO@CF and **(b,d,f)** Ag@ZnO@CF-2.5; field emission SEM (FESEM) images depicting **(g)** ZnO and **(h)** Ag@ZnO particles. The red arrows indicate Ag nanoparticles. Reproduced from Manna et al. (2015) with permission from the American Chemical Society.

Manna et al. (2015) reported a self-cleaning cotton fabrics material that exhibited antimicrobial activity, attributed to the presence of a uniform coating of Ag/ZnO NPs over the cotton fabric (CF). In that work, a polyamine-mediated mineralization process was carried out to prepare the final material (Ag/ZnO/CF), wherein poly(allylamine) was utilized as mineralizer as well as reducing agent for the preparation of Ag- and Zn-based NPs. In addition, poly(allylamine) also assisted to obtain uniform coating of the nanostructured materials onto the threads of fabric. Zinc/amine complex was formed due to the interaction of amine groups of poly(allylamine) with Zn(II) ions. Subsequently, this complex was exposed to 60°C that eventually led to the generation of ZnO NPs. The contribution of poly(allylamine) to the process of reduction in Ag(I) to Ag(0) was shown by UV–vis spectroscopic measurements, while SEM revealed the size of the obtained particles (150–250 nm), which were coated onto the individual threads of fabric (Figure 4). The antimicrobial activity of different coated cotton fabric samples was evaluated, and it was noticed that 100% reduction in viability was achieved for both *S. aureus* and *P. aeruginosa*. In another report, carbon nanofibers (CNFs) were developed on the substrate of activated carbon fiber (ACF) by employing chemical vapor deposition (CVD) toward development of carbon micro–nanofiber web comprising asymmetrical distribution of bimetallic Cu and Ag NPs. The Cu NPs were observed to catalyze CNF growth while positioned at the CNFs tips with the latter remaining attached on the surface of ACF. The obtained carbon micro–nanofiber web exerted lethal effects against *E. coli* and *S. aureus* in water for 7 days (Singh et al., 2014). In 2017, Ali et al. (2017) reported the antimicrobial activity of different combinations of bimetallic (Co/Ag, Co/Cu) NPs immobilized on carbon black–chitosan fibers (CB–CS) against *E. coli*. In that work, CB–CS fibers were dipped into solution of bimetal salts, and the immobilized metal ions on fibers were reduced using sodium borohydride (NaBH_4_). In the presence of *E. coli*, the obtained materials (Co/Ag–CB–CS) and (Co/Cu–CB–CS) exhibited growth inhibition rates of 91.1 and 92.7%, respectively. Jang et al. (2014) also reported a study based on CNFs loaded with Sn/ZrO_2_ NPs exerting lethal effects against *E. coli*. Sol–gel electrospinning was utilized for the formation of the CNFs–Sn/ZrO_2_ composite, and its successful synthesis was confirmed by XRD, revealing the presence of diffraction peaks of ZrO_2_ and Sn NPs, the former displayed at 2θ = 30, 35, 50, and 60° for the corresponding crystallographic planes of (111), (200), (220), and (311), while the latter showed the diffraction peaks associated with Sn tetragonal structure along with an overlapped diffraction peak at 30°, reflecting also the presence of SnO minor phase in the CNFs–Sn/ZrO_2_ composite. This resulting bimetallic composite exhibited enhanced antimicrobial activity against *E. coli* as compared to the monometallic analogs, a fact that is attributed to the synergistic effect of Sn and ZrO_2_ present in the matrix of CNFs.

Ashfaq et al. (2016) developed CNFs incorporated with asymmetrically dispersed Cu and Zn NPs (Cu/Zn–ACFs/CNFs) for antibacterial wound dressing applications. Chemical vapor deposition (CVD) was employed to design this material in such a way that CNFs were developed on ACFs with Cu NPs located at the tips of CNFs, while Zn NPs were distributed on the ACFs. Dosages of 1 and 2 mg/ml of Cu/Zn–ACFs/CNFs exerted significant antibacterial activity along with prolonged suppression of *E. coli, S. aureus*, and methicillin-resistant *S. aureus* (MRSA). Rapid release of Zn helped in the repair of body cells, while the slower release of Cu suppressed the growth of bacteria for a long period of time. Hence, the combination of rapid and slow release of Zn and Cu, respectively, enabled the generated carbon fiber composite to be utilized as antibacterial wound dressing material. The monometallic-based fibers could not exert analogous performance, as they lacked different time-release profiles of metals, unlike the bimetallic ones. In 2017, Ashfaq et al. (2017) reported another work for antibacterial wound dressing applications, wherein their previously reported material, i.e., Cu/Zn–ACFs/CNFs, was reinforced in the integrated matrix of polyvinyl alcohol (PVA) and cellulose acetate phthalate (CAP). The resulting PVA/CAP–Cu/Zn–ACF/CNF formulation exhibited substantial antimicrobial efficacy against *P. aeruginosa*. All reported fiber-supported bimetallic NPs with their corresponding preparation method, inhibition zone, MIC values, and targeted microbes are listed in Table 3.

**Table 3:** Types and antimicrobial properties of fiber-supported bimetallic nanoparticles.

**SR No**.	**Material**	**Method**	**Microbial strain**	**Inhibition zone (mm) or rate %**	**MIC (mg/ml)**	**Other performance indicators**	**References**
1	Ag/ZnO/CF-2.5	Bioinspired mineralization	*S. aureus*/*P. aeruginosa*	30/30	–	–	Manna et al., 2015
	Ag/ZnO/CF-5		*S. aureus*/*P. aeruginosa*	30/30			
2	Ag:Cu–ACF/CNF	CVD	*E. coli*/*S. aureus*	100%	–	–	Singh et al., 2014
3	Co/Ag–CB–CS	Chemical reduction	*E. coli*	91.1%	–	–	Ali et al., 2017
	Co/Cu–CB–CS			92.7%			
4	CNFs–Sn/ZrO_2_	Sol–gel electrospinning	*E. coli*	–	–	Antimicrobial kinetic test	Jang et al., 2014
5	Cu/Zn–ACFs/CNFs	CVD	*E. coli*/*S. aureus*/MRSA	–	1	–	Ashfaq et al., 2016
6	PVA/CAP–Cu/Zn–ACF/CNF	CVD and polymerization	*P. aeruginosa*	–	6	–	Ashfaq et al., 2017

### Polymer-Supported Bimetallic Nanoparticles

Boomi et al. (2013) applied a simple chemical methodology to synthesize pristine polyaniline (PANI) and polyaniline/Ag–Pt (PANI–Ag–Pt) materials, the antimicrobial properties of which were evaluated against Gram-positive and Gram-negative bacterial strains. It was revealed that PANI–Ag–Pt nanocomposite possessed superior antimicrobial efficacy over pristine polyaniline. The zones of inhibition of PANI–Ag–Pt for *Streptococcus* sp., *S. aureus, Klebsiella* sp. and *E. coli* bacteria were 29 ± 1.12, 30 ± 1.25, 25 ± 0.96 and 21 ± 0.95 mm, respectively. FTIR confirmed the presence of platinum and silver NPs in the synthesized nanocomposite. Arfat et al. (2017a) investigated the antimicrobial activity of fish skin gelatin (FSG) extracted biofilms and FSG biofilms reinforced with Ag–Cu NPs. The solution casting technique was applied to prepare FSG and FSG–Ag–Cu nanocomposite-based films. FTIR confirmed the association of NPs with gelatin matrix in the film. The films are potent antibacterial materials, as they inhibited activity against Gram-positive and Gram-negative strains. Bio-nanocomposite films are films that comprise biopolymers. Employing the obtained films as active food packaging material would prevent food damage from bacteria and other pathogens. It was revealed that loading of 1–4% Ag–Cu NPs showed better action against bacterial strains, while films loaded with 0.5% Ag–Cu NPs exerted no significant antibacterial activity. In another study, Boomi et al. (2014) examined the antimicrobial efficacy of PANI–Ag–Au nanocomposites. In that work, PANI–Ag–Au nanocomposite was prepared by performing chemical oxidative polymerization, and characterizations were executed via TEM, SEM, FTIR, etc. XRD validated the existence of both Au and Ag NPs in the matrix of PANI. Agar well-diffusion method was applied for evaluating the antimicrobial efficacy of the nanocomposites. PANI–Ag–Au exhibited substantial antimicrobial performance against both Gram-positive and Gram negative bacteria. It was inferred that PANI–Ag–Au possessed spherical morphology, which eventually resulted into antibacterial activity enhancement.

Arfat et al. (2017b) reported a study that included the synthesis of agar-based films incorporated with active particles by applying solution casting method and integrating 0.5–4.0 wt-% of Ag–Cu alloy NPs with glycerol plasticized agar solution. Characterization of the resulting nanocomposite films involved texture analysis, SEM, XRD, differential scanning calorimetry (DSC), FTIR, etc. XRD analysis revealed that the agar/Ag–Cu-based films possessed a crystalline structure. The obtained nanocomposite films were found to be promising as antibacterial agents against different bacterial strains; thus, they could be employed in the manufacturing of packaging material to preserve food against foodborne pathogens. The antimicrobial efficacy of the as-prepared films was estimated by liquid culture tests. Ahmed et al. (2018) studied plasticized polylactide (PLA) composite films synthesized by incorporating bimetallic Ag–Cu NPs and cinnamon essential oil (CEO) in the matrix of polymer by following a compression molding technique. The PLA composite films exhibited antimicrobial action against *L. monocytogenes, S. typhimurium*, and *Campylobacter jejuni*. It was noticed that the films loaded with combination of 4% NPs and 50% CEO exerted superior antibacterial performance. Boomi et al. (2013) reported another study based on antimicrobial property of PANI reinforced with bimetallic Au–Pd NP-based films. In that work, chemical reduction was applied to synthesize Au and Au–Pd colloids. PANI/Au- and PANI/Au–Pd-based films resulted from Au and Au–Pd colloidal solutions, while their synthesis was realized by chemical oxidative polymerization. It was observed that the antibacterial efficacy of PANI/Au–Pd surpassed that of PANI/Au against *E. coli, Staphylococcus* sp., *Streptococcus* sp. and *Klebsiella* sp. with corresponding values of inhibition zones of 25 ± 0.87, 22 ± 0.35, 21 ± 0.33, and 21 ± 0.31 mm, respectively.

Galashina et al. (2017) studied cellulose and polyester textile materials incorporated with mono- and heterometallic Cu and/or Ag sols to enhance the antimicrobial performance against microorganisms. Effective integration of chelators, such as amine derivatives of phosphonic acid, especially nitrilotrimethylenephosphonic acid (NTP), ethylenediamine tetra (methylene phosphonic acid) (EDTMP), and diethylenetriamine penta methylene phosphonic acid (DTPMP) in the formation of NPs was also demonstrated. Use of strong reducing agents, such as NaB*H*_4_, was involved in the synthesis of Cu NPs in aqueous solutions in order to reduce Cu cations. The introduction of gelatin additives further increased the stability of the sols. The advantage of embodied complex stabilizers in the formation of Cu and Cu–Ag NPs was revealed by spectrophotometry and AFM and PCS techniques. The synthesized cellulose/polyester–Cu–Ag NPs fibers were effective in providing antimicrobial activity against *C. albicans* fungus to natural and synthetic fibers and in protecting them from biodegradation by soil microflora and natural complex microflora. Hebeish et al. (2013) synthesized TiO_2_ nanowires (TiO_2_ NWs) by applying a hydrothermal method, whereas TiO_2_ NPs and NWs doped with Ag were also synthesized by photo-reducing Ag^+^ ions to Ag metal over the surfaces of the TiO_2_ NPs or TiO_2_ NWs. The resulting active materials were loaded on poly-*N*-vinyl-2-pyrrolidone (PVP)-treated cotton fabric for the evaluation of the antimicrobial activity against bacterial strains. Desired properties of wound dressing include anti-infection activity and skin regeneration. Hu et al. (2018) synthesized zinc oxide/silver/polyvinylpyrrolidone/polycaprolactone (ZnO/Ag/PVP/PCL) NPs by employing electrospinning. ZnO NPs are potent antibacterial and anti-inflammatory material that expedite the healing of acute and chronic wounds. ZnO/Ag/PVP/PCL exerted significant antibacterial action against *S. aureus* and *E. coli* bacteria. Enhancement of antimicrobial efficacy was noticed due to synergistic effect of the bimetallic nanofibers. Hassan et al. (2013) developed bimetallic ZnO–Ag immobilized in the matrix of polyurethane (PU) nanofibers (ZnO–Ag–PU) by applying electrospinning and sol–gel processing. The crystallinity of the nanofibers was examined through XRD, while antibacterial tests against *E. coli* bacterial strain were performed, revealing that ZnO–Ag–polyurethane nanofibers exhibited better antibacterial performance over pristine PU and ZnO–PU fibers. The distinctive ZnO and Ag NPs combination showed prominent bactericidal effects as a result of synergism. All reported polymer-supported bimetallic NPs with their corresponding preparation method, inhibition zone, MIC values, and targeted microbes are listed in Table 4.

**Table 4:** Types and antimicrobial properties of polymer-supported bimetallic nanoparticles.

**SR No**.	**Material**	**Method**	**Microbial strain**	**Inhibition zone (mm) or rate (%)**	**MIC (mg/ml)**	**Other performance indicators**	**References**
1	PANI–Ag–Pt film	Simple chemical method	*Streptococcus sp*./*S. aureus*/*Klebsiella sp*./*E. coli*	29/30/25/21	–	–	Boomi et al., 2013
2	FSG–Ag–Cu biofilms	Solution casting	*L. monocytogenes*/*Salmonella enterica* sv. *typhimurium*	–	–	Suppression of CFU ml^−1^	Arfat et al., 2017a
3	PANI–Ag–Au film	Chemical oxidative polymerization	*Streptococcus* sp. (MTCC 890)/*Staphylococcus* sp. (MTCC 96)	28/29/31/28	–	–	Boomi et al., 2014
			*E. coli*/(MTCC 1671)/*Klebsiella* sp. (MTCC 7407)				
4	Agar/Ag–Cu nanocomposites film	Solution casting method	*L. monocytogenes* (ATCC 19114)/*Salmonella enterica typhimurium* (ATCC 14028)	–	–	Suppression of CFU ml^−1^	Arfat et al., 2017b
5	PLA–Ag–Cu NPs film	Compression molding	*S. typhimurium*/*C. jejuni*/*L. monocytogenes*	–	–	Suppression of CFU ml^−1^	Ahmed et al., 2018
6	PANI–Au–Pd film	Chemical oxidative polymerization	*E. coli*/*Staphylococcus* sp./*Streptococcus* sp./*Klebsiella* sp.	25/22/21/21	25–150 μg/ml	–	Boomi and Prabu, 2013
7	Cellulose/polyester–Cu–Ag NPs fibers	Chemical reduction	*C. albicans*	–	–	–	Galashina et al., 2017
8	TiO_2_ NPs doped Ag−3% PVP-based cotton fabric	Hydrothermal method followed by immersion method	*P. aeruginosa*/*S. aureus E. coli*/*B. cereus*/*C. albicans*	19/15/17/15/13	–	–	Hebeish et al., 2013
	TiO_2_ NWs doped Ag-3% PVP based cotton fabric		*P. aeruginosa/S. aureus E. coli/B. cereus/C. albicans*	24/18/19/24/16			
9	ZnO/Ag/PVP/PCL	Electrospinning method	*S. aureus*/*E. coli*	–	–	–	Hu et al., 2018
10	ZnO–Ag–PU	Electrospinning method	*E. coli*	–	–	Suppression of CFU ml^−1^	Hassan et al., 2013

### Graphene-Supported Bimetallic Nanoparticles

**Figure 5:**
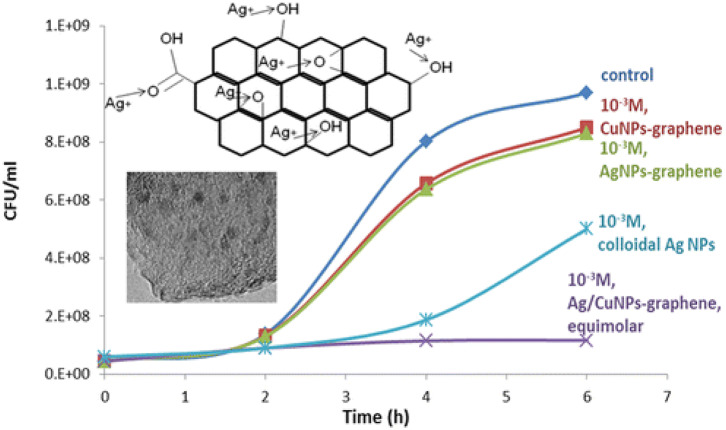
Enhanced antibacterial action of Ag/Cu bimetallic graphene hybrids. Reproduced from Perdikaki et al. (2016) with permission from the American Chemical Society.

Graphene is a two-dimensional material consisting of single or few layers of sp2-hybridized carbon atoms arranged in hexagonal lattice (Geim and Novoselov, 2010; Pilatos et al., 2014, 2016). The antimicrobial activity of graphene-supported bimetallic Ag–Cu nanocomposites was reported by Perdikaki et al. (2016). In that work, the antibacterial performance of the prepared hybrids was evaluated against *E. coli* cells and compared through a series of parameterization experiments of varying metal type and concentration. It was found that both Ag- and Cu-based monometallic graphene composites significantly suppressed bacterial growth, yet the Ag-based ones exhibited higher activity compared to that of their Cu-based counterparts. The synthesized hybrids were also compared with well-dispersed colloidal Ag NPs of same metal concentration, prepared in self-assembled amphiphilic block copolymer nanodomains in solution (Perdikaki et al., 2013). The Ag- and Cu-based graphene composites displayed weaker antibacterial activity than the respective colloidal NPs. Yet, the bimetallic Ag/CuNP–graphene hybrids exhibited superior performance compared to that of all other materials tested in that work, i.e., both the monometallic graphene structures as well as the colloidal NPs, achieving complete bacterial growth inhibition (Figure 5). This performance was attributed to the synergistic action of the combination of the two different metals that coexisted on the surface as well as the enhancing role of the graphene support. Subsequently, the work was further expanded with the development of graphene agents consisting of stabilized monometallic and bimetallic species in pure ionic form that exhibited an even higher antibacterial efficacy (Perdikaki et al., 2018).

**Figure 6:**
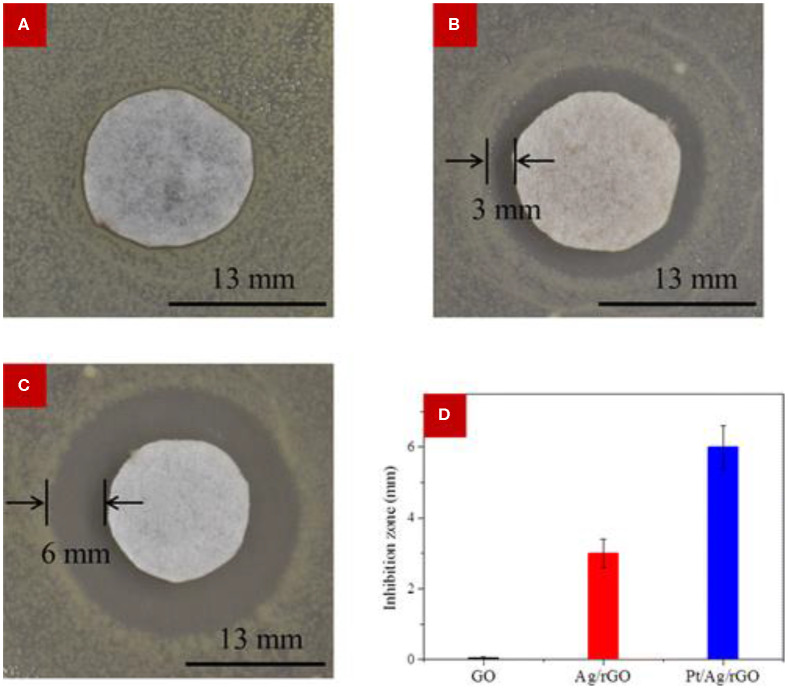
Antimicrobial activity measurement using the modified Kirby–Bauer method. **(A–C)** Optical images of bacterial colonies against *E. coli* corresponding to **(A)** GO, **(B)** Ag/rGO, and **(C)** Pt/Ag/rGO. The Pt/Ag/rGO sample exhibited the largest inhibition zone. **(D)** Comparisons of the radius of the inhibition zone of bacteria treated with the different samples, revealing superior performance of the bimetallic material. Error bar is based on the average of three measurements. Reproduced from Zhang et al. (2016a) with permission from the American Chemical Society.

Zhang et al. (2016a) investigated the antibacterial efficiency of reduced graphene oxide (rGO)-supported bimetallic Pt–Ag hybrids (Pt–Ag–rGO), and the activity against *E. coli* was compared with graphene oxide (GO) and monometallic Ag–rGO. It was observed that GO could not effectively suppress the growth of *E. coli*, displaying a radius of inhibition zone of 0.02 mm, while the growth of *E. coli* was effectively suppressed by Ag–rGO and Pt–Ag–rGO hybrid materials, with corresponding radii of zone of inhibition as 3 and 6 mm, respectively (Figure 6). Substantial antibacterial efficacy of hydrothermally prepared rGO–Ag–Fe_3_O_4_ nanocomposite against *E. coli* was reported by Joshi et al. (2014). The integration of Fe–Ag bimetallic NPs onto graphene sheet was also reported by Ahmad et al. (2016). In that report, the antimicrobial performance of the prepared graphene–Fe–Ag nanocomposite was carried out against bacterial strains of *E. coli, S. aureus*, and *B. subtilis*. A significant suppression of bacterial growth was achieved by Chen et al. (2015), who evaluated the antimicrobial activity of a Bi_2_WO_6_-graphene hybrid material, which was prepared by the surfactant-assisted hydrothermal method. A GO–TiO_2_-Ag hybrid material that involved two-dimensional GO sheet, one-dimensional TiO_2_ nanorods, and zero-dimensional Ag NPs was developed by a two-phase method and exerted high photocatalytic antibacterial activity against *E. coli* (Liu et al., 2013).

In 2017, Shanavas et al. (2017) demonstrated the antimicrobial performance of a nanocomposite material, which involved three different metals on rGO sheets. Specifically, La_2_CuO_4_-CeO_2_ and La_2_CuO_4_-CeO_2_-rGO hybrids were synthesized by solvothermal-assisted precipitation. The resulting hybrids suppressed significantly the growth of *E. coli* and *S. aureus*, displaying substantial inhibition zones at dosage of 100 μl. In another study, the solvothermal method was employed to develop graphene–MnFe_2_O_4_ nanocomposites, wherein bimetallic MnFe_2_O_4_ NPs were homogeneously distributed over the surface of graphene sheets, as revealed by SEM. The obtained material suppressed the *E. coli* growth by 82% (Chella et al., 2015). Cao et al. (2015) applied a microwave method for the development of a rGO–TiO_2_-MnO_*X*_ hybrid material. SEM and TEM images revealed that MnO_*X*_ dots and TiO_2_ NPs were present over the surface of rGO sheets, exerting superior antibacterial performance at different inhibition concentrations against *E. coli* and *S. aureus*. All reported graphene-supported bimetallic NPs with their corresponding preparation method, inhibition zone, MIC values, and targeted microbes are listed in Table 5.

**Table 5:** Types and antimicrobial properties of graphene-supported bimetallic nanoparticles.

**SR No**.	**Material**	**Method**	**Microbial strain**	**Inhibition zone (mm) or rate %**	**MIC (mg/ml)**	**Other performance indicators**	**References**
1	Graphene–Cu–Ag NPs	Wet chemical method (graphene by CVD)	*E. coli*	–	–	Suppression of CFU ml^−1^	Perdikaki et al., 2016
2	Cu–Ag ions stabilized on graphene	Solution method (graphene by CVD)	*E. coli*	–	–	Suppression of CFU ml^−1^	Perdikaki et al., 2018
3	Pt–Ag–rGO	Chemical method	*E. coli*	6 (radius)	–	–	Zhang et al., 2016a
4	rGO–Ag–Fe_3_O_4_	Hydrothermal	*E. coli*	–	–	–	Joshi et al., 2014
5	Graphene–Fe–Ag	Chemical reduction	*E. coli*/*S. aureus*/*B. subtilis*	>90%	–	–	Ahmad et al., 2016
6	Bi_2_WO_6_-graphene	Hydrothermal	–	–	–	Suppression of CFU ml^−1^	Chen et al., 2015
7	GO–TiO_2_-Ag	Two-phase	*E. coli*	–	–	Evaluation of bacterial viability	Liu et al., 2013
8	La_2_CuO_4_-CeO_2_	Solvothermal-assisted homogeneous precipitation	*E. coli*	24 ± 1.6	–	–	Shanavas et al., 2017
	La_2_CuO_4_-CeO_2_-rGO		*S. aureus*	22 ± 1.3			
			*E. coli*	26 ± 1.2			
			*S. aureus*	23 ± 1.5			
9	Graphene–MnFe_2_O_4_	Solvothermal	*E. coli*	82%	–	–	Chella et al., 2015
10	rGO–TiO_2_-MnO*_*X*_*	Microwave method	*S. aureus*	10.554	15.62 μg/ml	–	Cao et al., 2015
			*E. coli*	10.915	31.25 μg/ml		

### Non-supported Bimetallic Nanoparticles

Metal oxides like ZnO are known for their antibacterial properties. They absorb energy in the form of photons and produce reactive species like H_2_O_2_, superoxide, and hydroxyl radicals. These species are active, as they can break the outer shell of bacteria and inhibit their growth. Introducing metallic silver on the surface of ZnO generates a new energy level that can capture electrons excited in ZnO, thus preventing recombination. Ag doping on ZnO films has exerted antibacterial efficacy against *E. coli* bacteria both under UV light and in dark. The activity was significantly increased with silver loading due to the enhanced photocatalytic effect (Thongsuriwong et al., 2012). Zielinska-Jurek et al. (2015) studied the effect of metal NPs on TiO_2_ surface. Due to its large band gap (~3.2 eV), TiO_2_ can only be activated by UV light. Thus, the abundant sunlight source cannot be utilized. However, modification of TiO_2_ by plasmonic NPs (Pt, Ag, Au, and Pd) shifts the activity of wide band gap materials toward visible light due to the NP participation in the electron transfer mechanism. It was observed that bimetallic (Pt and Ag)-modified TiO_2_ (Ag–Pt–TiO_2_) synthesized by the sol–gel method exhibited enhanced antimicrobial activity in comparison to the monometallic form (Pt–TiO_2_/Ag–TiO_2_). The inhibition zones of Ag–Pt–TiO_2_ against *C. albicans, E. coli*, and *S. aureus* were respectively 9, 14, and 11 mm with corresponding MIC values of 16, >256, and >256 μg cm^−3^, respectively. In another report, Mittal et al. (2014) studied Ag–Se NPs, which were synthesized from quercetin and gallic acid at room temperature, for their antimicrobial activity against *E. coli* and *B. subtilis*. The inhibition zone of the resulting composite NPs against the two above bacteria types were 18 ± 0.5 and 19 ± 0.6 mm, respectively. More recently, Ag–SnO_2_ nanocomposites have attracted considerable attention due to their high activity, good stability, and lower cost compared to pure Ag. In this regard, Ag–SnO_2_ nanocomposites were synthesized with the aid of extracts of *Saccharum officinarum*. The extracts contained sugars, organic acids, and inorganic ions, which helped in the formation of the Ag–SnO_2_ nanocomposites. This material displayed antimicrobial action against *B. subtilis, E. coli, P. aeruginosa*, and *Streptococcus pneumoniae* (Sinha et al., 2017). CeO_2_ NPs are generally used for a number of biomedical applications. However, research works have provided mixed views regarding their antibacterial properties, with some suggesting antibacterial properties while others suggesting that this material does not exhibit antibacterial activity. In one study, reported by Masadeh et al. (2015), the antimicrobial efficacy of a combination of CeO_2_ and Fe_2_O_3_ NPs was investigated. The NPs were prepared via the hydrothermal route, yet the resulting materials were not noticeably active to inhibit growth of planktonic Gram-positive and Gram-negative bacterial cultures.

Dentistry requires dental restorative materials that can inhibit growth of microorganisms, a factor that is detrimental for dental health. In this regard, chemical routes were employed to synthesize Cu, Ni, and bimetallic Cu–Ni nanomaterials, and their antimicrobial efficacy was evaluated against human pathogens *S. aureus, E. coli*, and dental pathogen *S. mutans*. Cu NPs exhibited bactericidal effect against *E. coli, S. aureus*, and *S. mutans*, while Ni and Cu–Ni NPs exhibited almost similar effect against the same microorganisms (Argueta-Figueroa et al., 2014). In 2019, Antonoglou et al. (2019) reported the antimicrobial performance of Cu–FeO_2_ and Cu_2_O NPs, both synthesized by employing the hydrothermal method. The Cu–FeO_2_ NPs were found to be small crystalline particles with sizes of around 90–220 nm, while Cu_2_O NPs had a size of around 24.5 nm. Their antimicrobial activity was tested against *E. coli, B. subtilis, B. cereus, X. campestris*, and *S. aureus*. In the case of Cu–FeO_2_ NPs, the MIC values were recorded to be >100 μg/ml for all bacterial strains, while Cu–FeO_2_ NPs exerted very mild antimicrobial performance in comparison to Cu_2_O NPs. In another study, a chemical route was employed for the preparation of Au–Ag core–shell NPs. The NPs were investigated as antimicrobial agents and were employed for two photon excitation bacterial imaging (Ding et al., 2017). It was observed that aggregation of Au–Ag core–shell NPs bearing positive charge occurred onto the negatively charged surface of bacteria that eventually assisted in imaging due to enhanced 2PPL. These NPs exhibited activity against *S. aureus* with very less toxicity to human dermal fibroblasts. In another report of similar combination, Bankura et al. (2014) investigated the antimicrobial efficacy of Ag–Au alloy NPs, which were prepared by dextran as reducing agent at room temperature. The NP alloy was found to be stable, and it exerted considerable activity against *B. subtilis, B. cereus, E. coli*, and *P. aeruginosa* with corresponding inhibition zones of 24, 21, 17, and 20 mm at 0.1 mg/ml concentration of Ag–Au alloy NPs.

**Figure 7:**
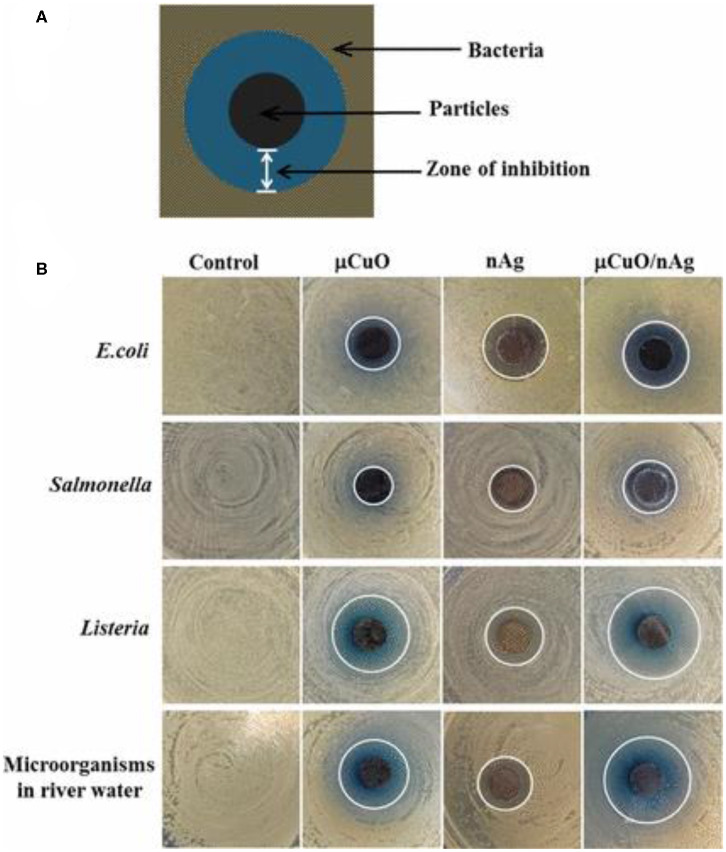
**(A)** Schematic diagram representative of the observed inhibition zone using bimetallic porous CuO decorated with Ag nanoparticles and **(B)** photographs of the inhibition zone with each particle type against Gram-negative bacteria *E. coli* and *Salmonella*, Gram-positive bacteria *Listeria*, and environmental microorganisms derived from river water. Note that the white lines mark the boundary of the inhibition zones; the control is a negative control without particles. Reproduced from Chen et al. (2017) with permission from the American Chemical Society.

Fe and Ag hybrid NPs also possess a high antimicrobial potential. Markova et al. (2013) explored such NP combinations for antibacterial and antifungal applications. Due to the magnetic properties of the Fe NPs, it was possible to control and remove them from place of action against fungi and bacteria by enabling magnetic-based separation. The preparation of these hybrid materials was achieved by reduction in Ag ions by commercially available zero-valent Fe NPs. Antimicrobial testing was realized by the microdilution method, which enabled the determination of MIC values. In another example of bimetallic combination, CuO–Ag NPs exhibited better antimicrobial efficacy against *E. coli, Salmonella, Listeria*, and other water-based microorganisms than that of the monometallic counterparts of CuO and Ag NPs. A bimetallic porous CuO/Ag complex was also synthesized by hydrothermal method followed by galvanic replacement. The antimicrobial action was found to be enhanced with increasing concentration for all synthesized monometallic and bimetallic materials. The corresponding inhibition zones were demonstrated against *E. coli, Salmonella, Listeria*, and water-based microorganism (Figure 7) (Chen et al., 2017). Zhang et al. (2016b) investigated the antibacterial activity of Ag-doped TiO_2_ nanowire (NW) arrays, synthesized by hydrothermal growth on sputtered AgTi layers that eventually exerted high antibacterial activity against *E. coli*. TEM and SEM imaging revealed that the obtained NW arrays possessed a crystalline phase and morphology having diameter and length of 85–95 nm and 11 μm, respectively. Andrade et al. (2017) synthesized Ag-decorated, star-shaped ZnO particles (Ag–ZnO NPs) using precipitation methods with assistance of thiourea (stabilizing agent). Then, the obtained precipitate was exposed to thermal treatment at 150°C. TEM images displayed a star-shape morphology of the Ag–ZnO nanostructure, wherein ZnO possessed a star-shape morphology, while Ag was attached to the ZnO surface in different morphologies, such as nanospheres, nanoplates, and nanorods. The synthesized –ZnO bimetallic NPs exhibited antimicrobial activity against *P. aeruginosa* (ATCC 27853) and *S. aureus* (ATCC 43300, ATCC 25923, and ATCC 33591), and their MIC values were determined to be around 16 μg/ml. Cai et al. (2017) synthesized porous Ag–Pt NPs by hydrothermal reduction, and the resulting bimetallic material showed high activity against *E. coli* and *S. aureus*. All non-supported bimetallic NPs reviewed herein with their corresponding preparation method, inhibition zone, MIC values, and targeted microbes are listed in Table 6.

**Table 6:** Types and antimicrobial properties of non-supported bimetallic nanoparticles.

**SR No**.	**Material**	**Method**	**Microbial strain**	**Inhibition zone (mm) or rate %**	**MIC**	**Other performance indicators**	**References**
1	Ag-doped ZnO NPs	Sol–gel dip coating	*E. coli*	–	–	Suppression of CFU ml^−1^	Thongsuriwong et al., 2012
2	Ag–Pt–TiO_2_ NPs	Sol–gel	*C. albicans*/*E. coli*/*S. aureus*	9/14/11	16/>256/>256 μg cm^−3^	–	Zielinska-Jurek et al., 2015
3	Ag–Se NPs	–	*E. coli*/*Bacillus subtilis*	18 ± 0.5/19 ± 0.6	–	–	Mittal et al., 2014
4	Ag–SnO_2_NPs	Precipitation	*B. subtilis*/*E. coli*/*P. aeruginosa*/*S. pneumoniae*	–	0.075/0.04/0.07/0.025 M	–	Sinha et al., 2017
5	CeO_2_-Fe_2_O_3_NPs	Hydrothermal	Planktonic bacterial cultures	NA	–		Masadeh et al., 2015
6	Cu–Ni NPs	Simple chemical method	*S. aureus*/*E. coli*/*S. mutans*	–	1,000 μg/ml	–	Argueta-Figueroa et al., 2014
7	Cu–FeO_2_ NPs	Hydrothermal	*E. coli*/*B. subtilis*/*B. cereus*/*X. campestris*/*S. aureus*	–	>100 μg/ml	–	Antonoglou et al., 2019
8	Au–Ag core–shell NPs	Simple chemical method	*S. aureus*	–	7.5 pM	–	Ding et al., 2017
9	Ag–Au alloy NPs	Wet chemical method	*B. subtilis*/*B. cereus*/*E. coli*/*P. aeruginosa*	24/21/17/20	0.1 mg/ml	–	Bankura et al., 2014
10	Fe–Ag NPs	–	Fungus and bacterial strains	–	100–540 mg/L	–	Markova et al., 2013
11	CuO–Ag NPs	Hydrothermal	*E. coli*/*Salmonella*/*Listeria*/water microorganism	–	50 μg/ml	–	Chen et al., 2017
12	Ag–TiO_2_ nanowires	Hydrothermal	*E. coli*	–	–	Suppression of CFU ml^−1^	Zhang et al., 2016b
13	Star-shaped Ag–ZnO NPs	Precipitation	*P. aeruginosa* (ATCC 27853)/*S. aureus* (ATCC 43300, ATCC 25923, and ATCC 33591)	–	15.63 μg/ml	–	Andrade et al., 2017
14	Ag–Pt NPs	Hydrothermal	*E. coli*/*S. aureus*	–	20 μg/ml	–	Cai et al., 2017

## Conclusions

In this work, developments in the relatively new area of combining more than one type of metals in nanoparticle formulations for antibacterial applications are reviewed. Both non-supported and support-assisted bimetallic systems have been examined, highlighting in particular the association between preparation methods, properties, and antimicrobial efficacy. The reported bimetallic NP systems exerted substantial antimicrobial activity against various microbial strains, and most of them were found to outperform their monometallic counterparts due to synergistic effects. The latter is attributed to the fact that each metal type can contribute through its own unique physicochemical characteristics and interaction potential with bacterial cells and with the other metal counterpart, as well as through different mechanisms of antibacterial action.

Conclusively, bimetallic NPs possess a promising potential as antimicrobial agents and have the tendency to exert significant contribution in several industries affected by microbes including water, food, textiles, and oil and gas. As such, further studies should be conducted in a systematic mode as to parametrically investigate growth of highly efficient agents based on bimetallic or multimetallic loadings, both in free-standing formulations or by employing properly engineered, high surface area porous supports able to bound various metallic species not only in nanoparticle but also in ionic forms. Targeted outcome of these studies should be the delivery of novel, efficient, and robust nanostructured systems able to significantly contribute toward battling the major problem of bacterial spread and antimicrobial resistance, including applicability in hospitals and in common areas. The developed materials should also be engineered to fit the targeted applications, be provided in various formulations, and be ready to be applied on various surfaces.

It should be highlighted, however, that upon preparation of such powerful NP-based antibacterial agents, and despite the fact that metal species can have the ability to distinguish bacterial from mammalian cells, it is an important prerequisite to investigate the biocompatibility and interaction effects with healthy cells of the designed nanosystems before application. Structure, size, solubility, ability to cross various biological barriers, aggregation within a system, generation of oxidants, ability to percolate into nucleus, exposure time, dosage, as well as pro-oxidant nature that can damage biomolecules and cell membranes are characteristics of such NPs that need to be examined by parallel toxicity studies before introducing them as efficient yet safe antimicrobial agents.

## Author Contributions

All authors listed have made a substantial, direct and intellectual contribution to the work, and approved it for publication. NA collected most of the literature, constructed all tables, and wrote the core section Bimetallic Nanoparticle Systems and Their Antimicrobial Properties. KT contributed to the writing of the sections Introduction, Antimicrobial Action Through Different Mechanisms and Synthesis Routes. GK supervised the work, wrote the abstract and conclusions, edited the manuscript, and attracted the funding.

## Conflict of Interest

The authors declare that the research was conducted in the absence of any commercial or financial relationships that could be construed as a potential conflict of interest.
